# Delayed visceral malperfusion caused by kinking of a four-branched integrated frozen elephant trunk graft

**DOI:** 10.1093/icvts/ivaf052

**Published:** 2025-03-01

**Authors:** Takuya Matsushiro, Tomoki Tamura, Nobuyuki Inoue, Tadashi Kitamura

**Affiliations:** Department of Cardiovascular Surgery, National Center for Global Health and Medicine, Tokyo, Japan; Department of Cardiovascular Surgery, National Center for Global Health and Medicine, Tokyo, Japan; Department of Cardiovascular Surgery, National Center for Global Health and Medicine, Tokyo, Japan; Department of Cardiovascular Surgery, Kitasato University School of Medicine, Sagamihara, Japan

**Keywords:** aortic dissection, four-branched frozen elephant trunk graft, graft kinking, TEVAR

## Abstract

An integrated frozen elephant trunk with a four-branched vascular graft may shorten the circulatory arrest time during open distal anastomosis. We report a case of acute type A aortic dissection where postoperative graft kinking led to acute liver failure, kidney injury and lower limb ischaemia corrected by thoracic endovascular aortic repair.

## INTRODUCTION

In January 2023, the Frozenix 4 Branched (Japan Lifeline Co., Ltd, Tokyo, Japan) became commercially available. This device integrates stented and non-stented grafts with branches, potentially reducing suturing time and circulatory arrest time during open distal anastomosis [1].

Frozen elephant trunk (FET) graft kinking is a potential complication, and graft kinking has been reported in the non-integrated Frozenix [2]. Here in, we describe our experience with intraoperative branched FET graft kinking.

## CASE PRESENTATION

A 56-year-old man presented to our emergency department with vital signs indicative of shock due to a cardiac tamponade. After pericardial drainage via the subxiphoid window, contrast-enhanced computed tomography (CT) revealed Stanford type A (type A E2 M0) acute aortic dissection. The intimal tear extended from the ascending aorta to the aortic arch. The aortic arch was classified as type II, with a distance of 13 mm from the brachiocephalic artery to its apex, equivalent to one to two times the diameter of the common carotid artery. The true lumen diameter of the proximal descending aorta was measured at 20 mm. The patient was immediately transferred to the operating room.

**Figure 1:**
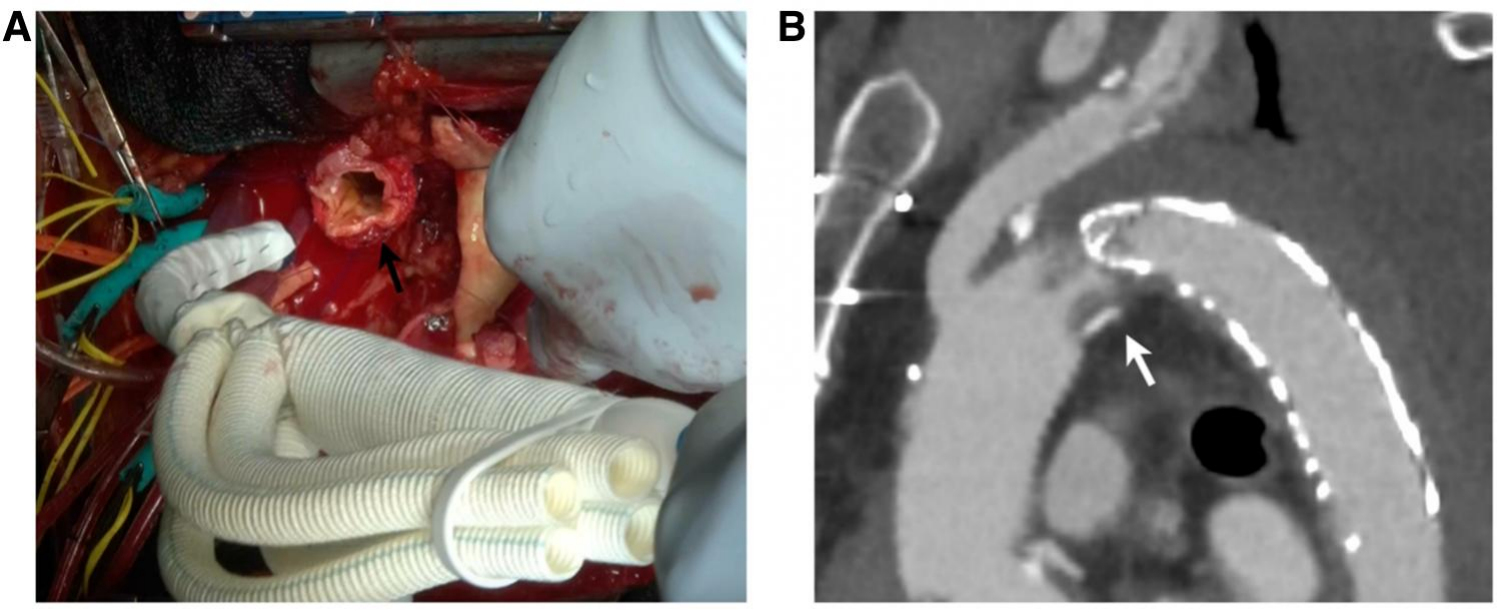
(**A**). Intraoperative images show small diameter of aortic arch (black arrow). (**B**) Postoperative CT angiography shows that the graft narrowed at the joint (white arrow).

Cardiopulmonary bypass was established via right atrial drainage, returning into the femoral artery, which is the standard cannulation approach for aortic dissection surgery at our institution. Under hypothermic (26°C) circulatory arrest with selective cerebral perfusion, the aortic arch was transected at zone 2. The diameters of the aortic arch and landing zone were 28 and 24 mm, respectively. A 25-mm, four-branched Frozenix with a 90-mm long stent was inserted distally (Fig. 1A). After stent deployment, the sewing cuff and native aortic wall were sutured, followed by total arch replacement.

**Figure 2:**
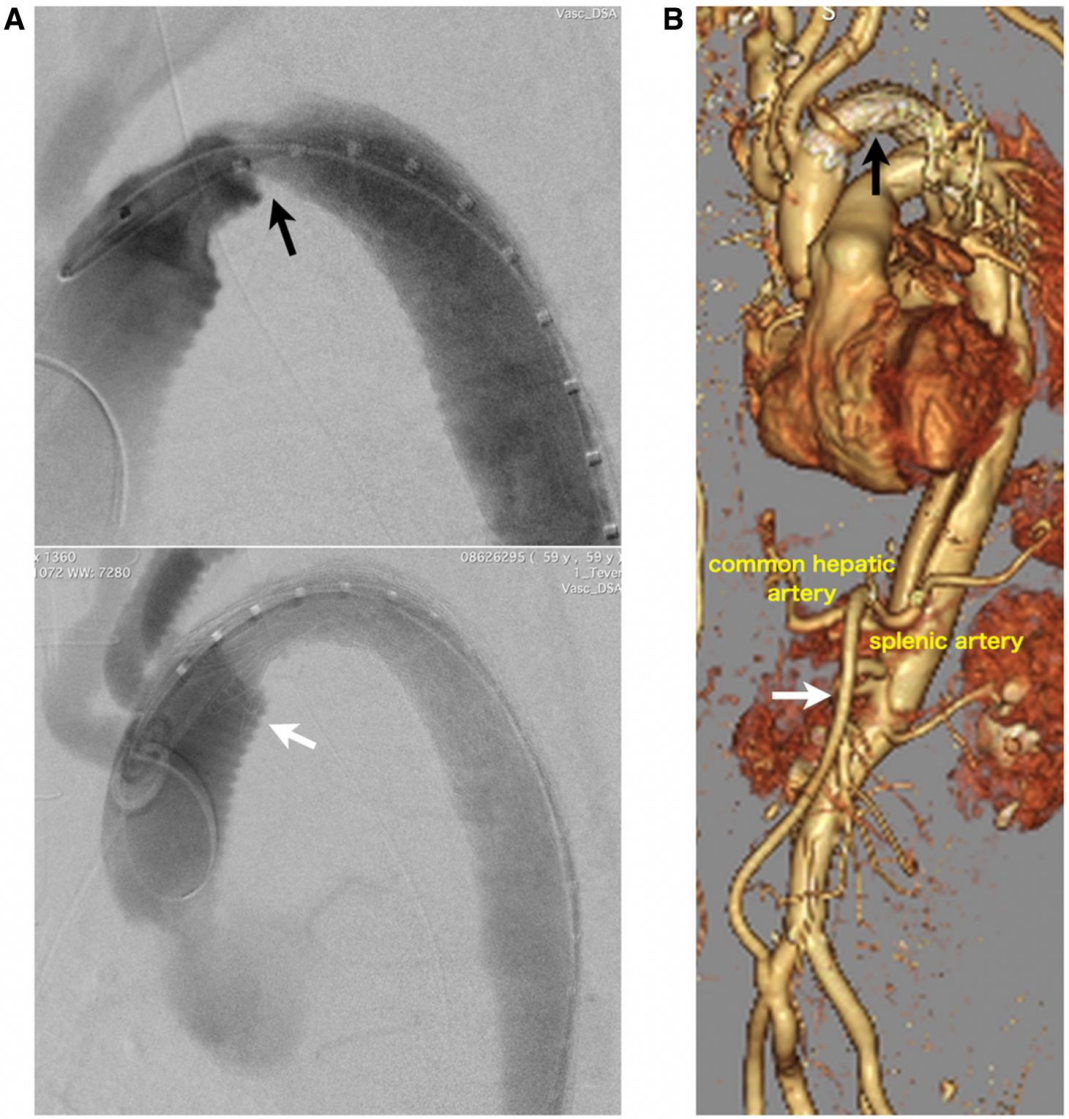
(**A**) Angiography conducted during thoracic endovascular aortic repair (TEVAR) indicates kinking of Frozenix graft at the junction (black arrow). The stent graft is located from the non-stented part (white arrow). (**B**) Post-TEVAR CT angiography shows that the kink was corrected (black arrow) and an ilio-coeliac artery bypass is placed (white arrow).

Two days later, the patient developed acute renal and liver failure with anuria and elevated serum creatinine, liver enzymes and bilirubin levels. Blood pressures in the upper and lower limbs were 140 and 60 mmHg, respectively. CT angiography revealed FET graft kinking at the boundary between the stented and non-stented sections (Fig. 1B), which caused coeliac artery malperfusion. Consequently, a 28 × 100 mm cTAG (Gore and Associates, Flagstaff, AZ, USA) was placed into the aortic arch following implantation of an iliohepatic artery bypass (Fig. 2A). Postoperatively, lower body circulation, including that of the renal artery, improved (Fig. 2B). Liver enzymes and serum creatinine levels subsequently normalized. The patient’s postoperative course was mostly uneventful, except for a concomitant cerebral infarction that required long-term rehabilitation. After discharge, he was referred to another hospital to continue rehabilitation. To date, no long-term complications related to the graft have been observed.

## DISCUSSION

The FET procedure enables a one-stage extensive replacement of the ascending aorta, aortic arch and zone 4 descending aorta through a median sternotomy. In Japan, the use of FET for acute type A aortic dissection increased from 15% in 2016 to 29% in 2021 [3]. The four-branched Frozenix comprises a woven polyester graft with a 15-mm turned-up sewing cuff and a 90-mm long stent graft, which has a nitinol stent sewn 5 mm distal to the connection, creating a 20 mm non-stented portion inserted into the aorta that serves as an adequate landing zone.

Previous reports have indicated that intraoperative kinking of the conventional Frozenix occurred at the junction between the stented and non-stented sections, which was attributed to the excessive length of the non-stented section [2]. As a countermeasure, the length of the non-stented section should be minimized. However, since the length of the non-stented section in the four-branched Frozenix was fixed, this adjustment was impossible. Other reports mentioned that kinking is more likely in younger patients with a highly angled aortic isthmus [4]. In our case, stenosis still occurred at the graft junction despite the aortic arch not being acutely angled and the stented section covering the aortic isthmus.

This case involved the use of a FET graft for acute aortic dissection in a narrow true lumen, potentially contributing to stenosis. In contrast to the conventional two-piece design, it is difficult to visually confirm from inside the lumen whether the stented section has fully expanded. In the future, we plan to use an aortic balloon to expand the stented and non-stented sections within the aorta to prevent kinking and stenosis. The Frozenix includes a nitinol stent that features an endoskeletal structure inside the graft. Compared with other FET devices, its expansion force is slightly weaker, which helps prevent distal stent-induced new entry. This characteristic suggests that postexpansion using a balloon can be safely performed.

## Data Availability

All relevant data are within the manuscript.
